# Policy implications of regional variations in eye disease detection and treatment on Prince Edward Island: a repeated cross-sectional analysis, 2010–2012

**DOI:** 10.1186/s12913-018-3068-z

**Published:** 2018-04-10

**Authors:** A. M. Khan, G. E. Trope, R. Wedge, Y. M. Buys, S. El-Defrawy, Q. Chen, Y. P. Jin

**Affiliations:** 1 Dalla Lana School of Public Health, University of Toronto, 155 College St, Toronto, ON M5T 3M7 Canada; 2 Department of Ophthalmology and Vision Sciences, University of Toronto, 340 College Street, Suite 400, Toronto, ON M5T 3A9 Canada; 3 Health PEI, 16 Garfield Street, Charlottetown, PEI C1A 7N8 Canada; 4 University of Waterloo, 200 University Ave W, Waterloo, ON N2L 3G1 Canada

**Keywords:** Vision care coverage policy, Eye care utilization, Glaucoma, Cataracts, Diabetes

## Abstract

**Background:**

In Canada, government insurance covers eye care services provided by ophthalmologists and other physicians. However, government coverage for services provided by optometrists, non-medical school trained primary eye care providers, varies regionally. Little is known about the impact of a funding model in which ophthalmologist services are government-insured but services provided by optometrists are not, on eye care utilization and eye disease detection and treatment. We aimed to address this question by examining geographic variations in eye care service utilization on Prince Edward Island (PEI).

**Methods:**

PEI physician-billing data from 2010 to 2012 was analyzed across five distinct geographic regions (Charlottetown, Summerside, Prince, Queens & Kings and Stratford). The residential location of patients and practice locations of eye care providers were identified using the first three digits of their respective postal code. Age-standardized rates were computed for comparisons across different regions.

**Results:**

There were six ophthalmologists practicing on PEI, five with offices in Charlottetown. Twenty optometrists practiced on the island with offices across the province. Stratford is closest and Prince farthest from Charlottetown. Age-standardized utilization rates of ophthalmologists per 100 populations were 10.44 in Charlottetown and 10.90 in Stratford, which was significantly higher than in other regions (7.74–8.92; *p* < 0.05). The disparities were most pronounced amongst the elderly. The prevalence of glaucoma visits was higher in Charlottetown (6.10%) and Stratford (6.38%) and lower in other regions. A similar pattern was observed for the prevalence of cataract visits. While the prevalence of diabetes visits was higher in Prince and Summerside, the utilization of ophthalmologists by people with diabetes was almost twice as high in Charlottetown (6.49%) than in Prince (3.88%).

**Conclusions:**

The observed discrepancies in vision care utilization across geographic regions were likely attributed to barriers in accessing government-insured, geographically concentrated ophthalmologists, as opposed to a reflection of the true differences in eye disease occurrence. The lower prevalence of glaucoma visits in regions farther away from ophthalmologist offices may result in delayed detection and blindness in this population. Encouraging ophthalmologists to work in other areas of the province and/or to publicly fund services provided by optometrists may mitigate the observed disparities.

**Trial registration:**

Not applicable.

## Background

In publicly funded healthcare systems such as in Canada, Sweden, France and the United Kingdom, cost-related barriers to accessing healthcare services, including eye care services, should theoretically be averted. In Canada, despite public funding, the model for delivery of healthcare, including eye care, varies by region. This is because there are 13 different provincial/territorial health insurance plans [1]. With financial assistance and guidance from the Federal government, each Canadian provincial and territorial government designs, manages and funds healthcare services for their residents [1]. Consequently, a harmonized nationwide healthcare, or vision care coverage policy does not exist. As a result, some Canadian provinces cover eye care services provided by ophthalmologists, other physicians and optometrists whilst others insure services provided by ophthalmologists and other physicians only [2–6].

Optometrists are non-medical school educated primary eye care providers who are trained and equipped to provide eye care services, including prescribing eyeglasses and contact lenses. They also manage and treat patients with mild to moderate eye diseases and refer those with significant medical or surgical concerns to ophthalmologists [7]. Ophthalmologists are specialized medical doctors providing both medical and surgical eye/vision care [8].

In some Canadian provinces, and in countries such as the Netherlands where optometric services are not publicly funded, the costs associated with visiting an optometrist have to be paid out-of-pocket, through employment insurance, or a mix of both [9]. Although provision of services by ophthalmologists are publicly funded in all Canadian jurisdictions, ophthalmologists generally require a referral letter from a healthcare professional such as an optometrist or a family physician before they provide the service. Additionally, both the number and geographic distribution of practicing ophthalmologists is smaller than that of practicing optometrists [10]. One study reported that there were 3.35 ophthalmologists vs. 16.48 optometrists per 100,000 Canadians [10]. Furthermore, of the 148 Canadian census areas, only one area, Yellowknife, did not have an optometrist but 43 (29%) areas had no ophthalmologist [10]. Thus, for many people, accessing eye care services provided by an ophthalmologist can be challenging not only because of the requirement of a referral letter but also the physical distance to the nearest office, while obtaining optometric services may be difficult due to the out-of-pocket costs incurred by the patient.

Little is known about whether there exists unequal detection and treatment of eye disease based on an individual’s residential location in the context of a funding model where services provided by ophthalmologists are publicly funded but services provided by optometrists are not. Using Prince Edward Island (PEI) as an example, we aimed to address this knowledge gap by examining geographic variations in the clinical assessment and management of diabetic eye exams, glaucoma and cataracts. PEI is a province in Canada where prior to August 2015, the government did not insure any eye care services provided by optometrists [11].

## Methods

### Data sources and study regions

**Fig. 1:**
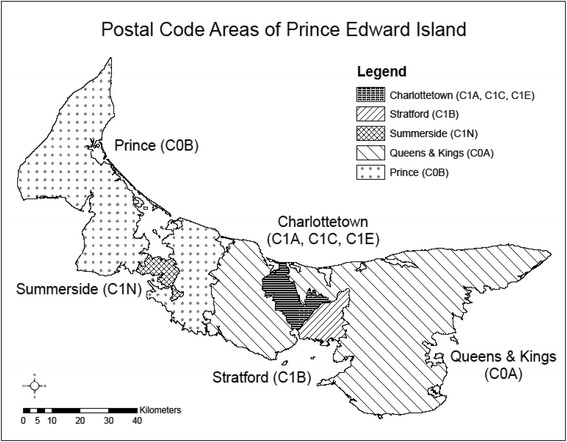
The five geographic regions of study interest located on Prince Edward Island, Canada. Regions were defined based on a distinctive forward sortation area, which is defined as the first three digits of the postal code. Forward Sortation Area boundary data obtained from Statistics Canada, Forward Sortation Area boundary data, 2011. This does not constitute an endorsement by Statistics Canada of this product

PEI physician billing data, a population-based database containing information on all physician claims for services provided through the provincial medical care plan, from 2010 to 2012 was analyzed across five geographic regions. The five regions were Charlottetown (“C1A,” “C1C,” “C1E”), Summerside (“C1N”), Prince (“C0B”), Queens & Kings (“C0A”) and Stratford (“C1B”) (Fig. 1). These regions were selected owing to their distinct forward sortation areas (FSA), which is the first 3 digits of the postal code.

Three vision-related conditions managed by ophthalmologists were examined: diabetes, glaucoma and cataracts. These diseases were selected because they 1) represent a major source of eye disease burden in the population, 2) visits related to these conditions would be government funded for all ages, and 3) these diseases can be readily recognized in physician billing data using diagnostic codes. The physician billing data utilized in this study was collected primarily for government billing purposes and as such, the diagnostic codes in the database may not necessarily represent a definitive diagnosis or a true presence of that condition, but a patient visit for diagnosis, treatment or follow-up examinations of that condition. The three vision-related conditions identified by related International Classification of Diseases, 9th revision (ICD-9) codes were assigned as a glaucoma (365) visit, cataract (366) visit or diabetes (250) visit. Ophthalmologist services were recognized using physician specific specialty codes.

If a patient had multiple visits for the same condition in a given study year, only one (i.e., the latest) visit was included in the analysis. Similarly, if a patient had multiple visits to an ophthalmologist in a particular year, only the latest visit was counted. The practice site of ophthalmologists was located and mapped using their clinic postal code. Patient residential location was determined by their residential FSA.

### Statistical analyses

Prevalence rates of eye conditions were calculated as the region and year specific total counts divided by the population counts in that region in 2011 as reported in Statistics Canada’s census data [12]. At the time of the study, the most recent age-specific population numbers for all the geographic regions of interest were publicly available from the 2011 Census data. Prevalence rates were directly age-standardized to the 2011 PEI Census population (i.e., the standard population) to allow for comparisons across regions, accounting for their differing age structures. The utilization of ophthalmologists was similarly computed. Standardized rate ratios (SRR), obtained by dividing one age-standardized rate by another, and their corresponding 95% confidence intervals (CIs) were calculated to assess the statistical significance of difference in standardized rates [13]. Statistical significance was assessed at the *p* < 0.05 level.

All analyses were conducted using SAS version 9.4 (SAS Institute, Cary NC). The geographic maps were produced using ArcGIS version 10.0 (Esri, Redlands CA). Ethics approval for this study was obtained from the PEI Research Ethics Board.

## Results

### Study regions and practice locations of ophthalmologists and optometrists

**Fig. 2:**
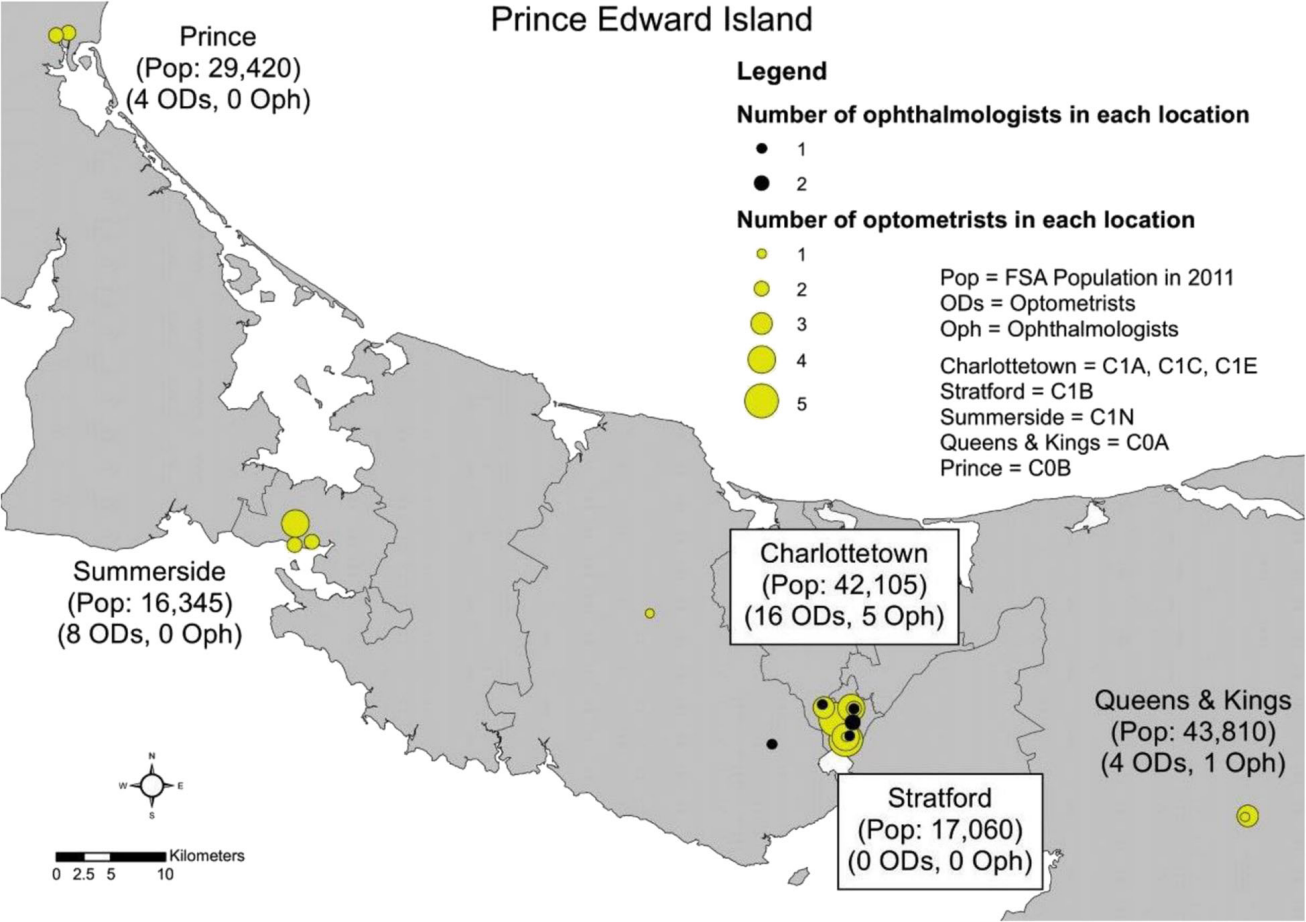
Numbers of practicing ophthalmologists and optometrists in addition to their practice locations, on Prince Edward Island, Canada in 2014. FSA, forward sortation area; OD, optometrists; Oph, ophthalmologists; pop, 2011 population in the forward sortation area. Regions were defined based on distinctive forward sortation areas, defined as the first three digits of the postal code. Each dot represents a location where an optometrist or ophthalmologist practices with some optometrists or ophthalmologists having multiple clinic locations. Forward Sortation Area boundary data and population counts obtained from Statistics Canada, Forward Sortation Area boundary data, 2011. This does not constitute an endorsement by Statistics Canada of this product

During the study period there were a total of six ophthalmologists on PEI, 5 (83%) with offices located in Charlottetown and one in the city of Cornwall (Fig. 2). Cornwall is located approximately 11 km west of the city Charlottetown, belonging to the region Kings & Queens. In contrast, there were 20 optometrists practicing on the island, with offices located across the province.

Of the five regions examined, Stratford is closest and Prince farthest from Charlottetown (Fig. 1). Since optometric services were not government insured during the study period, 76,000 people, or about 54% of islanders residing outside the Greater Charlottetown Area, would have had to travel to Charlottetown, or its neighbouring city Cornwall, to receive provincially funded eye care services provided by ophthalmologists [14–16].

### Prevalence of eye diseases and utilization of ophthalmologists

In the year 2012, the age-standardized utilization rate of ophthalmologists per 100 persons was 10.44 (95% CI: 10.13, 10.75) in Charlottetown and 10.90 (95% CI: 10.16, 11.64) in Stratford (Table 1).

**Table 1:** Age-standardized rates (per 100 population) and standardized rate ratios for diabetes-related visits, ophthalmologists visits and ophthalmologist visits amongst people with diabetes in those of all ages, Prince Edward Island, Canada, 2010–2012

Year; region	Diabetes visits			Ophthalmologist visits			Ophthalmologist visits amongst those with diabetes		
	Number of visits	Rate (95% CI)^a^	SRR (95% CI)	Number of visits	Rate (95% CI)^a^	SRR (95% CI)	Number of visits	Rate (95% CI)^a^	SRR (95% CI)
2010									
Charlottetown^¶^	2157	5.18 (4.96, 5.40)	0.92 (0.87, 0.98)	3743	8.97 (8.68, 9.26)	Ref.	227	7.18 (5.69, 8.67)	Ref.
Summerside^§#^	1035	5.93 (5.56, 6.29)	1.06 (0.98, 1.14)	1275	7.15 (6.75, 7.54)	0.80 (0.75, 0.85)	74	4.30 (2.90, 5.70)	0.60 (0.42, 0.86)
Prince^§#^	1668	5.62 (5.35, 5.88)	Ref.	1999	6.70 (6.40, 6.99)	0.75 (0.71, 0.79)	71	4.20 (2.32, 6.08)	0.58 (0.38, 0.90)
Kings & Queens^¶§^	2128	4.89 (4.68, 5.10)	0.87 (0.82, 0.93)	3273	7.58 (7.32, 7.84)	0.84 (0.81, 0.89)	197	8.51 (6.36, 10.66)	1.18 (0.85, 1.65)
Stratford^¶^	354	4.55 (4.07, 5.02)	0.81 (0.73, 0.90)	665	8.62 (7.96, 9.28)	0.96 (0.89, 1.04)	44	7.05 (4.42, 9.67)	0.98 (0.64, 1.50)
2011									
Charlottetown^¶^	2520	6.05 (5.81, 6.29)	0.91 (0.86, 0.97)	4347	10.41 (10.10, 10.72)	Ref.	288	7.18 (5.82, 8.53)	Ref.
Summerside^§#^	1223	7.01 (6.62, 7.41)	1.06 (0.98, 1.14)	1419	7.98 (7.56, 8.39)	0.77 (0.72, 0.81)	80	3.79 (2.69, 4.89)	0.53 (0.38, 0.73)
Prince^§#^	1972	6.64 (6.34, 6.93)	Ref.	2316	7.75 (7.44, 8.07)	0.74 (0.71, 0.78)	88	4.02 (2.46, 5.59)	0.56 (0.38, 0.82)
Kings & Queens^¶§^	2554	5.88 (5.65, 6.10)	0.89 (0.83, 0.94)	3700	8.57 (8.29, 8.84)	0.82 (0.79, 0.86)	231	7.15 (5.65, 8.66)	1.00 (0.75, 1.32)
Stratford^¶^	411	5.28 (4.77, 5.80)	0.80 (0.72, 0.88)	802	10.37 (9.65, 11.10)	1.00 (0.92, 1.07)	52	7.97 (5.25, 10.69)	1.11 (0.74, 1.66)
2012									
Charlottetown^¶^	2490	5.97 (5.74, 6.21)	0.86 (0.81, 0.91)	4361	10.44 (10.13, 10.75)	Ref.	274	6.49 (5.24, 7.74)	Ref.
Summerside^§#^	1165	6.65 (6.27, 7.03)	0.96 (0.89, 1.03)	1457	8.19 (7.77, 8.61)	0.78 (0.74, 0.83)	79	3.92 (2.77, 5.08)	0.60 (0.43, 0.84)
Prince^§#^	2060	6.93 (6.63, 7.23)	Ref.	2310	7.74 (7.43, 8.06)	0.74 (0.71, 0.78)	91	3.88 (2.31, 5.44)	0.60 (0.40, 0.89)
Kings & Queens^¶§^	2646	6.10 (5.87, 6.33)	0.88 (0.83, 0.93)	3848	8.92 (8.64, 9.20)	0.85 (0.82, 0.89)	246	8.21 (6.42, 10.00)	1.27 (0.94, 1.70)
Stratford^¶^	423	5.44 (4.92, 5.97)	0.79 (0.71, 0.87)	842	10.90 (10.16, 11.64)	1.04 (0.97, 1.13)	49	6.79 (4.45, 9.12)	1.05 (0.70, 1.56)

*Abbreviations*: *CI* confidence intervals, *Ref* reference, *SRR* Standardized rate ratio

Regions were defined based on distinctive forward sortation areas (first 3 digits of the postal code)

^a^Rates were age-standardized to the 2011 Prince Edward Island population

^¶^ *P* < 0.05 for diabetes related visits compared to Prince

^§^ *P* < 0.05 for ophthalmologist visits compared to Charlottetown

^#^ *P* < 0.05 for ophthalmologist visits amongst those with diabetes compared to Charlottetown

**Fig. 3:**
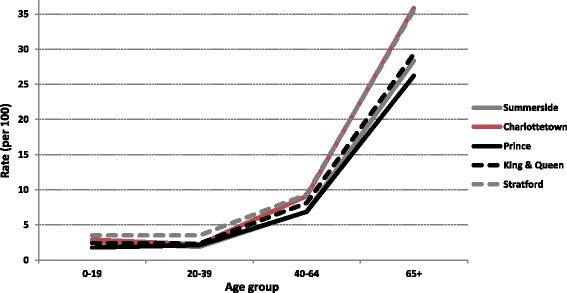
Age-specific utilization rates (per 100 populations) of ophthalmologist across the five study regions on Prince Edward Island, Canada in 2012. Regions were defined based on a distinctive forward sortation area, defined as the first three digits of the postal code

Relative to Charlottetown, utilization rates observed in the other regions were significantly lower (Summerside: SRR 0.78, 95% CI: 0.74–0.83; Prince: SRR 0.74, 95% CI: 0.71–0.78; King & Queens: SRR 0.85, 95% CI: 0.82–0.89) (Table 1). Age-specific rates revealed the greatest regional disparities in visits to ophthalmologists were amongst those aged 65+ (Fig. 3).

The prevalence of glaucoma visits per 100 people aged ≥40 years in 2012 was higher in Charlottetown (rate: 6.10, 95% CI: 5.78, 6.42) and Stratford (rate: 6.38, 95% CI: 5.59, 7.17) and lower in the other regions. The lowest age-standardized rates were observed in Prince (rate: 3.85, 95% CI: 3.55, 4.16), with the prevalence of glaucoma visits being 37% lower in Prince compared to Charlottetown (SRR 0.63, 95% CI: 0.58–0.69) (Table 2).

**Table 2:** Age-standardized rates (per 100 populations) and standardized rate ratios for glaucoma-related visits in people aged 40 years and older, Prince Edward Island, Canada, 2010–2012

Year; region	Number of visits	Rate (95% CI)^a^	SRR (95% CI)
2010			
Charlottetown	1255	5.59 (5.28, 5.90)	Ref.
Summerside	427	4.35 (3.94, 4.76)	0.78 (0.70, 0.86)
Prince	591	3.66 (3.37, 3.96)	0.66 (0.60, 0.72)
Kings & Queens	1032	4.47 (4.20, 4.74)	0.80 (0.74, 0.87)
Stratford	218	5.45 (4.73, 6.18)	0.98 (0.85, 1.13)
2011			
Charlottetown	1368	6.09 (5.77, 6.42)	Ref.
Summerside	445	4.56 (4.13, 4.98)	0.75 (0.68, 0.83)
Prince	618	3.83 (3.53, 4.13)	0.63 (0.57, 0.69)
Kings & Queens	1142	4.95 (4.66, 5.23)	0.81 (0.75, 0.88)
Stratford	245	6.17 (5.39, 6.94)	1.01 (0.88, 1.16)
2012			
Charlottetown	1370	6.10 (5.78, 6.42)	Ref.
Summerside	455	4.62 (4.19, 5.05)	0.76 (0.69, 0.84)
Prince	622	3.85 (3.55, 4.16)	0.63 (0.58, 0.69)
Kings & Queens	1198	5.20 (4.90, 5.49)	0.85 (0.79, 0.92)
Stratford	253	6.38 (5.59, 7.17)	1.05 (0.91, 1.20)

*Abbreviations*: *CI* confidence intervals, *Ref* reference, *SRR* Standardized rate ratio

Regions were defined based on distinctive forward sortation areas (first 3 digits of the postal code)

^a^Rates were age-standardized to the 2011 Prince Edward Island population

*p* > 0.05 for rates in Stratford compared to Charlottetown for all years; *p* < 0.05 for rates in Summerside, Kings & Queens and Prince compared to Charlottetown in all years

The prevalence of cataract visits was similarly higher in Charlottetown and lower in Prince and other regions (Table 3). Cataracts rates were significantly lower in Prince than Charlottetown (SRR 0.76, 95% CI: 0.69–0.84).

**Table 3:** Age-standardized rates (per 100 populations) and standardized rate ratios for cataract-related visits in people aged 40 years and older, Prince Edward Island, Canada, 2010–2012

Year; region	Number of visits	Rate (95% CI)^a^	SRR (95% CI)
2010			
Charlottetown	769	3.41 (3.17, 3.65)	Ref.
Summerside	306	3.06 (2.72, 3.40)	0.90 (0.79, 1.02)
Prince	418	2.59 (2.34, 2.83)	0.76 (0.68, 0.85)
Kings & Queens	629	2.76 (2.54, 2.97)	0.81 (0.73, 0.90)
Stratford	116	2.96 (2.42, 3.50)	0.87 (0.72, 1.04)
2011			
Charlottetown	1000	4.43 (4.15, 4.70)	Ref.
Summerside	362	3.62 (3.25, 3.99)	0.82 (0.73, 0.92)
Prince	549	3.40 (3.11, 3.68)	0.77 (0.69, 0.85)
Kings & Queens	746	3.27 (3.04, 3.51)	0.74 (0.67, 0.81)
Stratford	144	3.72 (3.11, 4.33)	0.84 (0.71, 0.99)
2012			
Charlottetown	1017	4.50 (4.22, 4.78)	Ref.
Summerside	344	3.43 (3.07, 3.79)	0.76 (0.68, 0.86)
Prince	555	3.43 (3.15, 3.72)	0.76 (0.69, 0.84)
Kings & Queens	779	3.43 (3.19, 3.67)	0.76 (0.69, 0.84)
Stratford	166	4.29 (3.63, 4.94)	0.95 (0.81, 1.12)

*Abbreviations*: *CI* confidence intervals, *Ref* reference, *SRR* Standardized rate ratio

Regions were defined based on distinctive forward sortation areas (first 3 digits of the postal code)

^a^Rates were age-standardized to the 2011 Prince Edward Island population

*P* < 0.05 for rates in Prince and Kings & Queens compared to Charlottetown in 2010, 2011 and 2012; p < 0.05 for rates in Summerside compared to Charlottetown in 2011 and 2012, but not 2010; *p* > 0.05 for rates in Stratford compared to Charlottetown for all years examined except 2011

The prevalence of diabetes visits per 100 persons was higher in Prince (6.93) and Summerside (6.65) and lower in the other regions (range: 5.44–6.10; *p* < 0.05 compared to Prince) in 2012 (Table 1). While assessment, diagnosis and treatment of diabetes do not require a visit to an ophthalmologist, guidelines recommend that those with diabetes should visit an ophthalmologist regularly for an eye check-up [17]. We observed that the age-standardized utilization rate of ophthalmologists amongst people with diabetes was approximately twice as high in Charlottetown (rate: 6.49 per 100, 95% CI: 5.24, 7.74) and Stratford (rate: 6.79 per 100, 95% CI: 4.45, 9.12) compared to that in Prince (3.88 per 100) and Summerside (3.92 per 100) (Table 1). Amongst those with claims for diabetes-related visits, those residing in Summerside and Prince were 40% less likely to visit an ophthalmologist (Summerside: SRR 0.60, 95% CI: 0.43–0.84; Prince: SRR 0.60, 95% CI: 0.40–0.89).

Similar trends were observed for all outcomes reported above in the years 2010 and 2011.

## Discussion

The provision of universal healthcare in PEI and other jurisdictions in Canada is aimed at helping Canadians access healthcare services, including eye care services, without concerns regarding their ability to pay [18]. As such, one would expect a fairly equitable utilization of healthcare providers and services within a population. However, we report large geographic variations in eye care services provided on PEI. Specifically, we found significantly higher utilization rates of ophthalmologists and higher prevalence of glaucoma, cataract and diabetic eye care visits for Canadians residing in Charlottetown and its neighbouring region Stratford. The lowest rates were observed in Prince, which is also farthest from the region of Charlottetown. Since the prevalence of diabetic visits was found to be higher in Prince, but visits to ophthalmologists amongst those with diabetes-related visits lowest in Prince, it is less likely that the reported disparities are a reflection of true differences in eye disease occurrence, but are more likely to be attributed to differences in access to government-insured, geographically concentrated ophthalmologist offices.

In the largely privatized United States (U.S.) healthcare system, lack of health insurance is frequently cited as a barrier to eye care utilization [19–21]. In Canada, health insurance, or the lack thereof, is generally not a concern for eye care access because all provincial health insurance plans cover eye care services, provided that the patient has a medically diagnosed eye disease. However, our study reveals that despite having a publicly funded healthcare system on PEI, marked geographic disparities occur in eye care utilization and eye disease detection and treatment. This indicates that universal health coverage is, in of itself, not enough to reduce inequalities in access to healthcare services. Other factors such as travel times, absence from work and travel costs to the clinic may also be important factors in a patient’s decision to see an ophthalmologist. For example, Alberton, a city in Prince, is 122 km from Charlottetown, requiring travel times of approximately 1.5 h by car. This issue is further complicated by the fact that there are no public transportation options available. Similar geographic barriers have previously been cited as reasons for inequitable healthcare access and outcomes [22, 23]. Such barriers may be particularly pronounced for seniors, the poor and patients with vision problems. Geographic barriers may be responsible for the greater disparities in ophthalmologist visits observed amongst the oldest patient group in this study.

The prevalence of glaucoma in the present study differs from rates observed in the literature. A 2004 meta-analysis of population-based studies conducted in the U.S., Australia, and Europe reported the overall prevalence of primary open-angle glaucoma, a specific type of glaucoma, in the U.S. population ≥ 40 years to be 1.86% [24]. Similar findings were observed in the Beaver Dam Eye Study [25]. In Canada, the prevalence of self-reported glaucoma of any type was 2.7% in 2002/2003, with a trend towards increasing rates from 1994/1995 to 2002/2003 [26]. The prevalence of glaucoma visits in our study (3.85%–6.38%) was higher than reported in the literature. Several reasons may account for these differences. First, our analyses included any patient visit related to glaucoma (e.g., diagnostic assessment, treatment and follow-up of any type of glaucoma) while other studies included only those patients diagnosed with primary open-angle glaucoma. Second, it is possible that rates of glaucoma are higher in PEI than in other regions. This is supported by data from the Canadian Community Health Survey (CCHS), which reported glaucoma rates amongst Caucasian Canadians aged 40+ to be higher on PEI (4.1%), compared to Ontario (2.6%) and the national average (2.6%). Lastly, each of these studies examined different time periods, with rising secular trends in glaucoma prevalence rates reported in recent years [26]. As such, one may expect the prevalence of glaucoma rates computed using data collected in 2010–2012 to be greater than the prevalence rates reported from data collected in the 1990’s or early 2000’s.

Based on clinic examinations, the prevalence of late-stage cataracts in the Beaver Dam Eye Study in the U.S. ranged from 5.5% for people aged 55–64 to 52.2% for those aged 75–84, while the Blue Mountains Eye Study in Australia reported prevalence rates of late cataracts of 2.7% for people aged 43–54 and 67.9% for those aged 85+ [27, 28]. The prevalence rates of cataracts in our study were 3.43%–4.50% in 2012. These rates are likely not a true reflection of the prevalence of cataracts in the province, as cataract diagnoses by an optometrist would not have been captured in the provincially funded billing data utilized in this study.

As noted in this study, optometrists are distributed across PEI, while ophthalmologists’ practices are concentrated in the capital. These findings are reflective of trends nationwide [10, 29]. Utilization rates of ophthalmologists, as ascertained from the PEI billing data in our study, ranged from 6.70% to 10.90% across the five regions during the study period. This is significantly lower than the self-reported utilization of eye care providers (38.9%) by PEI residents’ aged 12+ in the 2010 CCHS, which included services provided by optometrists and ophthalmologists. This large discrepancy between utilization of government insured ophthalmologists reported in this study and the self-reported utilization of ophthalmologists and optometrists in the CCHS suggests that more than 66% of eye care services on PEI were provided by optometrists.

Encouraging ophthalmologists to work in underserved or rural areas through the provision of financial incentives or developing comprehensive teleophthalmology programs has been proposed as potential solutions which may afford patients, particularly those residing in remote areas, to more readily and conveniently access eye care providers. Such a funding model should be aimed at enhancing the outreach of eye care services and improving eye disease detection for residents in rural areas. While this financial incentive has been in place on PEI, it does not seem to have worked very well for eye care concerns [30]. In August 2015, the PEI government started to fund optometric services for three eye conditions, namely dry eye, red eye and diabetic eye screening [11]. This policy change affords a valuable opportunity in future studies to assess whether regional disparity in diabetic eye exams has been mitigated. Nonetheless, many other eye conditions served by optometrists are still not funded. The findings of the present study offer support to the PEI government to consider funding more optometric services to reduce geographic disparities in vision care.

Our study has some limitations. Firstly, the validity of the speciality and ICD-9 codes are unknown. Due to the strict payment schedules in place by the government, we believe the speciality coding for ophthalmologists is valid. It is unclear whether cases we identified through ICD-9 codes truly have the disease. While this may make comparisons with other studies difficult, as case definitions may vary, it does not invalidate the observed disparities across the five regions on PEI as the same case definitions were similarly employed across all regions. Lastly, we were unable to account for patients receiving care from optometrists because the database did not capture such services. It is also plausible that residents outside of Charlottetown received eye care from alternate publicly funded sources such as primary care practices and emergency departments. This is problematic given that eye diseases require specialized care. This problem is highlighted in a study conducted by Huang and colleagues which reported that most (85%) of the primary care physicians who claimed to routinely screen for glaucoma said they would refer the patient to an ophthalmologist or optometrist if they suspected the condition [31]. As a result, these alternative sources of care alone are unlikely to adequately address the reported eye care disparities caused by geographic residence. Future studies are needed to examine this possibility.

## Conclusions

In summary, we reported significantly higher utilization rates of ophthalmologists and a higher number of visits for glaucoma and cataracts related visits amongst PEI residents living in Charlottetown and its neighbouring region Stratford, compared to other regions. The lowest numbers were documented in regions (e.g., Prince) farthest from Charlottetown. It is likely that the reported patterns are attributable to a combination of ophthalmologist offices being concentrated in Charlottetown and the barriers associated with traveling long distances in order to receive provincially insured eye care services located primarily in Charlottetown. Encouraging ophthalmologists to work in other areas, developing teleophthalmology programs and improving the recently introduced public funding model for services to optometrists who work in all areas of PEI have the potential to narrow the access gap observed in the present study. Replicating the study in other Canadian provinces with a similar funding structure is warranted.

## Abbreviations

CCHS: Canadian Community Health survey
CI: Confidence intervals
FSA: Forward sortation area
ICD-9: International Classification of Diseases 9th revision
PEI: Prince Edward Island
SRR: Standardized Rate Ratios
U.S.: United States

## References

[1] Government of Canada (2016). Canada’s health care system.

[2] Ontario Ministry of Health and Long-term Care (2013). OHIP coverage for eye care services.

[3] Programs and Services for Seniors, Gouvernement du Québec: Optometric services (n.d.). http://www4.gouv.qc.ca/EN/Portail/Citoyens/Evenements/aines/Pages/services-optometriques-couverts.aspx. Accessed 17 Feb 2016.

[4] Alberta Health: What is covered under the AHCIP? (n.d.). http://www.health.alberta.ca/AHCIP/what-is-covered.html. Accessed 17 Feb 2016.

[5] Department of Health and Community Services, Newfoundland and Labrador (2017). Medical care plan coverage.

[6] New Brunswick: Coverage and Claims-Inside New Brunswick (n.d.). http://www2.gnb.ca/content/gnb/en/departments/health/MedicarePrescriptionDrugPlan/content/medicare/CoverageandClaimsInsideNewBrunswick.html. Accessed 17 Feb 2016.

[7] PEI Association of Optometrists: About PEIOA (n.d.). http://www.peioptometrists.ca/about-peioa/. Accessed 11 August 2017.

[8] Canadian Ophthalmological Association: What is Ophthalmology? (n.d.) http://www.cos-sco.ca/vision-health-information/what-is-ophthalmology/. Accessed 11 August 2017.

[9] Wammes J, Jeurissen P, Westert G. The Dutch health system, 2014. n.d. http://www.nvag.nl/afbeeldingen/Netherlands%20Health%20Care%20System%202014%20(PDF).pdf. Accessed 26 Feb 2017.

[10] Al Ali A, Hallingham S, Buys YM (2015). Workforce supply of eye care providers in Canada: optometrists, ophthalmologists, and subspecialty ophthalmologists. Can J Ophthalmol.

[11] Health PEI (2015). Islanders to have better access to eye care through their optometrist.

[12] Profile C (2016). Statistics Canada.

[13] Boyle P, Parkin DM, Jensen OM, Parkin DM, MacLennan R, Muir CS, Skeet RG (1991). Statistical methods for registries. Cancer registration: principles and methods.

[14] City of Charlottetown: Charlottetown Profile (n.d.). http://www.city.charlottetown.pe.ca/charlottetownprofile.php. Accessed 22 Nov 2016.

[15] Statistics Canada (2016). Focus on geography series, 2011 census.

[16] Statistics Canada (2015). Focus on Feography series, 2011 census.

[17] Hooper P, Boucher MC, Cruess A, Dawson KG, Delpero W, Greve M (2012). Canadian ophthalmological society evidence-based clinical practice guidelines for the management of diabetic retinopathy. Can J Ophthalmol.

[18] Health Canada (2012). Canada’s health care system.

[19] Zhang X, Saaddine JB, Lee PP, Grabowski DC, Kanjilal S, Duenas MR (2007). Eye care in the United States: do we deliver to high-risk people who can benefit most from it?. Arch Ophthalmol.

[20] Zhang X, Lee PP, Thompson TJ, Sharma S, Barker L, Geiss LS (2008). Health insurance coverage and use of eye care services. Arch Ophthalmol.

[21] Lee DJ, Lam BL, Arora S, Arheart KL, McCollister KE, Zheng DD (2009). Reported eye care utilization and health insurance status among US adults. Arch Ophthalmol.

[22] Wilson K, Rosenberg MW (2002). The geographies of crisis: exploring accessibility to health care in Canada. Can Geogr.

[23] Cloutier-Fisher D, Penning MJ, Zheng C, Druyts E (2006). The devil is in the details: trends in avoidable hospitalization rates by geography in British Columbia, 1990-2000. BMC Health Serv Res.

[24] Friedman DS, Wolfs RC, O’Colmain BJ, Klein BE, Taylor HR, West S (2004). Prevalence of open-angle glaucoma among adults in the United States. Arch Ophthalmol.

[25] Klein BE, Klein R, Sponsel WE, Franke T, Cantor LB, Martone J (1992). Prevalence of glaucoma. The beaver dam eye study. Ophthalmology.

[26] Perruccio AV, Badley EM, Trope GE (2007). Self-reported glaucoma in Canada: findings from population-based surveys, 1994-2003. Can J Ophthalmol.

[27] Klein BE, Klein R, Linton KL (1992). Prevalence of age-related lens opacities in a population. The beaver dam eye study. Ophthalmology.

[28] Mitchell P, Cumming RG, Attebo K, Panchapakesan J (1997). Prevalence of cataract in Australia: the Blue Mountains eye study. Ophthalmology.

[29] Bellan L, Buske L, Wang S, Buys YM (2013). The landscape of ophthalmologists in Canada: present and future. Can J Ophthalmol.

[30] Medical Society of Prince Edward Island, Government of Prince Edward Island, Health PEI. Master Agreement between The Medical Society of Prince Edward Island and The Government of Prince Edward Island and Health PEI. n.d. http://www.gov.pe.ca/photos/original/doh_masteragree.pdf. Accessed 28 Nov 2016.

[31] Huang JT, Rhemtulla F, Huang PT (2003). Glaucoma screening by primary care physicians in southern Alberta: patterns, methods and deficiencies. Can J Ophthalmol.

